# Factors Associated with Diagnosis-Based Incident Mild Cognitive Impairment in Middle-Aged Women: A 14-Year National Cohort Study

**DOI:** 10.3390/healthcare14213701

**Published:** 2026-11-01

**Authors:** JooHee Kim, ChaeWeon Chung

**Affiliations:** 1 College of Nursing, Eulji University, Uijeongbu-si 11759, Republic of Korea; 2 Research Institute of Nursing Science, College of Nursing, Seoul National University, Seoul 03080, Republic of Korea; chungcw@snu.ac.kr

**Keywords:** comorbidity, mild cognitive impairment, middle-aged women, missing data, reproductive factors

## Abstract

Background/Objectives: This study aimed to identify sociodemographic, physiological, lifestyle-related, reproductive, and comorbidity factors associated with diagnosis-based incident mild cognitive impairment (MCI) among middle-aged Korean women and to descriptively compare findings from complete-case analysis (CCA) with results from five separately imputed datasets. Methods: This retrospective cohort study used customized data from the Korean National Health Insurance Service from 2009 to 2022. The source population comprised women aged 40–65 years who underwent general and cancer health screenings during 2009–2010. After eligibility and disease-related exclusions and removal of duplicate records, 2,178,195 women remained before missing-data handling. CCA included 483,184 women, of whom 34,976 met the definition of diagnosis-based incident MCI during 2011–2022. Diagnosis-based incident MCI was operationally defined as Korean Standard Classification of Diseases code F06.7 recorded as the primary or first secondary diagnosis on at least two separate healthcare dates. Five imputed datasets were generated from the dataset before listwise deletion and analyzed separately without Rubin’s-rule pooling. Multivariable logistic regression was used to examine associated factors. Longitudinal-change variables compared measurements from 2009–2010 and 2021–2022 and were interpreted as associated factors rather than temporally established predictors. Results: In the CCA, associations with diagnosis-based incident MCI were observed for age, changes in employment status, BMI, physical activity, selected reproductive characteristics, and several comorbidities. Results from the five separately imputed datasets showed somewhat different patterns for several socioeconomic, physiological, lifestyle-related, reproductive, and comorbidity factors. Because the five imputed datasets were analyzed separately without Rubin’s-rule pooling, these findings were interpreted descriptively rather than as pooled multiple-imputation estimates. An exploratory sensitivity analysis using a broader composite cognitive and neurological outcome showed generally similar directions and magnitudes for several major associations. Conclusions: Diagnosis-based incident MCI in middle-aged women was associated with multidimensional health characteristics, including reproductive and comorbidity-related factors. However, differences between the CCA and results from the five separately imputed datasets cannot be attributed solely to missing-data handling and should be interpreted in light of differences in analytic procedures, temporal limitations, and substantial missingness.

## 1. Introduction

Mild cognitive impairment (MCI) is characterized by cognitive decline greater than expected with normal aging while functional independence in everyday activities is relatively preserved and is considered an intermediate clinical state between normal cognitive aging and dementia [1]. Although individuals with MCI are at increased risk of developing dementia, progression is not inevitable. The clinical course of MCI is heterogeneous; some individuals remain cognitively stable, whereas others revert to normal cognition. Recent meta-analyses have shown that reversion from MCI to normal cognition occurs in a substantial proportion of individuals, particularly in population-based studies [2]. This heterogeneous course underscores the importance of identifying MCI and associated health factors before more advanced cognitive decline occurs.

In Korea, opportunities for systematic identification of cognitive impairment during midlife remain limited. Cognitive impairment screening within the National Health Screening Program has primarily targeted older adults [3]. Midlife is also a period during which physiological changes and chronic health conditions relevant to subsequent cognitive health become increasingly apparent [4,5]. An approach focused primarily on cognitive impairment in older age may therefore miss an earlier period during which health characteristics relevant to cognitive health and MCI could be identified.

This issue is particularly relevant to middle-aged women. The menopausal transition is accompanied by changes in estrogen exposure that may influence neural processes involved in cognitive function. Longitudinal studies have demonstrated changes in memory and processing speed across the menopausal transition [6,7], and recent evidence has suggested an association between age at menopause and subsequent cognitive function [8]. The health implications of menopause also extend to cardiovascular, skeletal, and metabolic health. Korean women have a longer life expectancy than Korean men; in 2024, life expectancy at birth was 86.6 years for women and 80.8 years for men [9]. These menopausal and broader health transitions, together with longer life expectancy, highlight the importance of addressing cognitive health in middle-aged women.

MCI in middle-aged women cannot be adequately understood by considering individual factors in isolation. Factors associated with MCI span sociodemographic, physiological, lifestyle-related, and chronic health domains. Reproductive characteristics—including age at menarche and menopause, parity, breastfeeding, hormone replacement therapy, and oral contraceptive use—represent an additional dimension of women’s health reflecting reproductive and hormonal experiences across the life course. A comprehensive examination of sociodemographic, physiological, lifestyle-related, reproductive, and comorbidity-related factors may therefore provide a more integrated understanding of MCI in middle-aged women. Such a multidimensional perspective may also be relevant to comprehensive health assessment by integrating lifestyle, reproductive, physical, and psychological health characteristics.

Large-scale National Health Insurance data enable these multidimensional factors to be examined simultaneously but also present challenges related to missing data. Complete-case analysis (CCA) excludes participants with missing information and may substantially reduce the analytic sample when missingness is extensive [10,11]. Multiple imputation (MI) uses observed information to impute plausible values for missing observations under specified assumptions, thereby retaining information that would otherwise be excluded [10,12]. Consequently, missing-data handling may affect not only the analytic sample but also the factors identified as being associated with MCI. This issue is particularly relevant when multiple health domains with differing degrees of missingness are examined simultaneously.

Accordingly, this study aimed to identify factors associated with diagnosis-based incident MCI among middle-aged women using a 14-year national cohort. In contrast to previous studies that have often examined individual health domains separately, this study considered sociodemographic, physiological, lifestyle-related, reproductive, and comorbidity factors within the same national cohort. In addition, findings from the complete-case analysis were descriptively compared with those from five separately imputed datasets to examine whether the pattern of observed associations differed across analytic pathways. By integrating female-specific reproductive characteristics with broader multidimensional health factors, this study offers a more comprehensive population-based perspective on cognitive health in middle-aged women.

## 2. Materials and Methods

### 2.1. Study Design

This retrospective cohort study used customized data from the National Health Insurance Service (NHIS) of Korea from 2009 to 2022. Factors associated with diagnosis-based incident mild cognitive impairment (MCI) among Korean women who entered the cohort during midlife were examined using complete-case analysis (CCA), with descriptive comparison to results from five separately imputed datasets. This study was reported in accordance with the Strengthening the Reporting of Observational Studies in Epidemiology (STROBE) guidelines.

### 2.2. Data Source and Participants

Data were obtained from four components of the NHIS customized database: eligibility and insurance contribution, general health screening, cancer screening, and healthcare utilization databases. The source population comprised Korean women aged 40–65 years who underwent both general health and cancer screenings during 2009–2010 and were followed through to 2022. Diagnoses were ascertained using the Korean Standard Classification of Diseases (KCD).

Women with MCI (F06.7), dementia (F00, F01, or F03), or Alzheimer’s disease (G30) at baseline were excluded. Dementia and Alzheimer’s disease were identified using diagnostic codes in conjunction with claims for anti-dementia medications, including donepezil, galantamine, rivastigmine, and memantine. At baseline, participants with predefined cerebrovascular or neurological disorders, including subarachnoid hemorrhage (I60), intracerebral hemorrhage (I61), other nontraumatic intracranial hemorrhage (I62), cerebral infarction (I63), stroke not specified as hemorrhage or infarction (I64), other cerebrovascular diseases (I67), cerebrovascular disorders in diseases classified elsewhere (I68), intracranial injury (S06), or Parkinson’s disease, were also excluded. Participants with dementia, Alzheimer’s disease, cerebrovascular disease, Parkinson’s disease, or other prespecified neurological disorders recorded on at least two separate healthcare dates during 2011–2022 were excluded from the analytic cohort. This exclusion criterion was applied across the entire follow-up period and did not distinguish whether the qualifying neurological diagnosis occurred before or after diagnosis-based incident MCI was identified. Accordingly, the resulting analytic cohort was not constructed to evaluate subsequent progression from MCI to dementia. Because these exclusions were based partly on events occurring after baseline, they may have introduced selection into the analytic cohort.

Among 2,789,523 potentially eligible women, 65,799 who died during 2009–2022 and 301,019 with predefined dementia, Alzheimer’s disease, cerebrovascular or neurological disorders, or Parkinson’s disease recorded on at least two separate healthcare dates during 2011–2022 were excluded. After removal of duplicate records (n = 244,510), 2,178,195 participants remained before missing-data handling.

**Figure 1:**
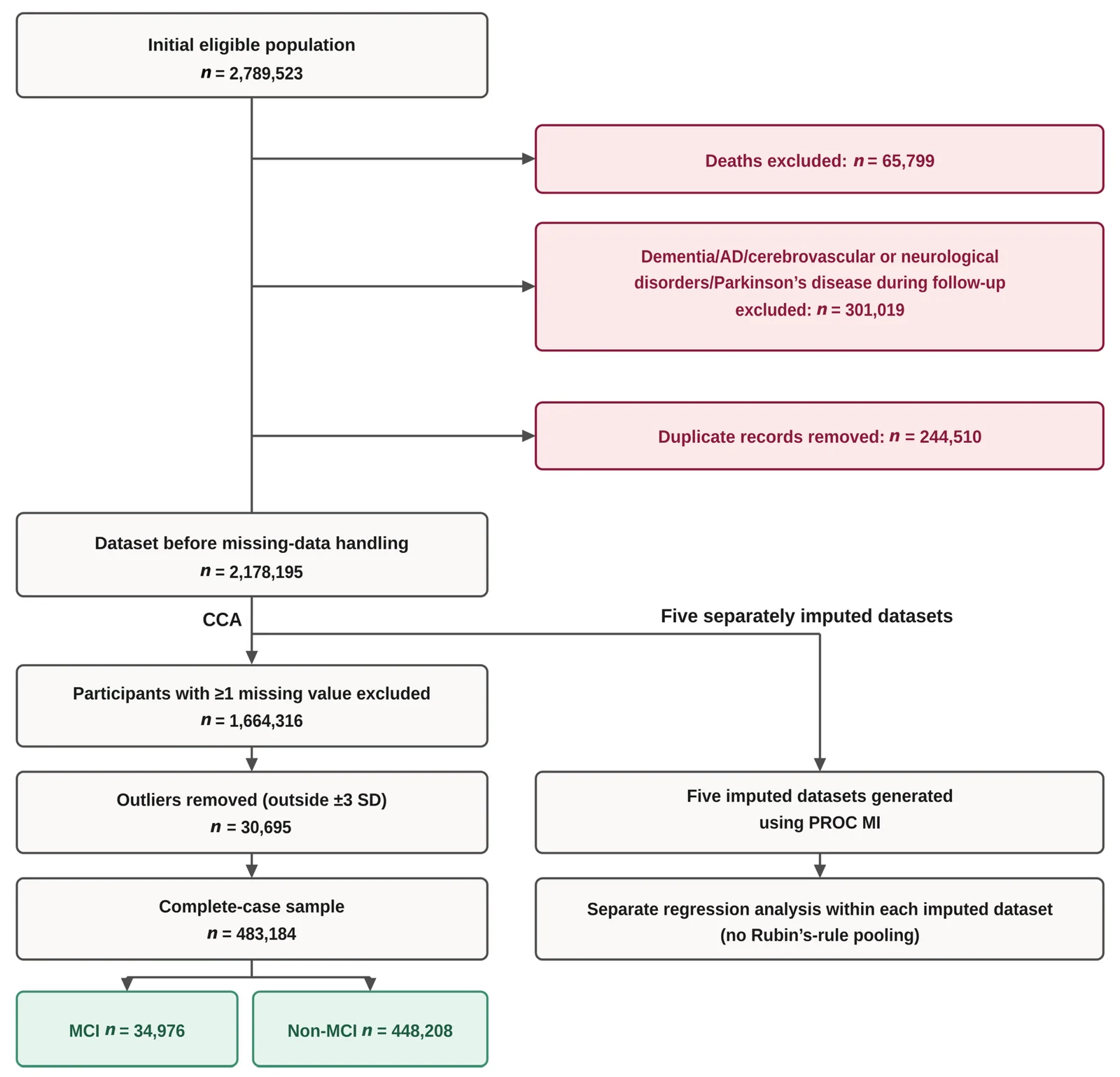
Flowchart of participant selection and analytic pathways for the complete-case analysis and five separately imputed datasets. Note: AD, Alzheimer’s disease; CCA, complete-case analysis; MCI, mild cognitive impairment; MI, multiple imputation; SD, standard deviation. After eligibility and disease-related exclusions and removal of duplicate records, 2,178,195 women remained before missing-data handling. For CCA, participants with at least one missing value in any variable included in the analysis were excluded, followed by exclusion of those with outlying values beyond ±3 SD from the variable-specific mean. For the analysis pathway involving the five separately imputed datasets, five imputed datasets were generated using PROC MI in SAS from the dataset before listwise deletion and were analyzed separately without Rubin’s-rule pooling.

For CCA, 1,664,316 participants with at least one missing value were excluded. In the complete-case analytic pathway, statistical outliers were screened for BMI, hemoglobin, and total cholesterol measured in 2009–2010 and 2021–2022, as well as age at menarche and age at menopause. For each variable, values more than three standard deviations below or above the variable-specific mean were considered statistical outliers. Participants with at least one value meeting this criterion were excluded (n = 30,695), resulting in a complete-case sample of 483,184 women, including 34,976 with diagnosis-based incident MCI and 448,208 without MCI. In contrast, the five imputed datasets were generated from the dataset before complete-case exclusion, and the ±3 SD outlier-exclusion procedure was not applied to this pathway. Five imputed datasets were generated using PROC MI in SAS version 9.4 (SAS Institute Inc., Cary, NC, USA). Participant selection and the complete-case and separately imputed dataset analytic pathways are presented in Figure 1.

### 2.3. Outcome: Diagnosis-Based Incident Mild Cognitive Impairment

Diagnosis-based incident MCI was operationally defined using diagnostic information recorded in the NHIS healthcare database. KCD code F06.7, which corresponds to mild cognitive disorder, was retained across KCD-6, KCD-7, and KCD-8 during the study period. F06.7 has also been used to identify MCI in previous Korean nationwide claims-based studies, although operational algorithms have varied across studies [13,14]. Women with F06.7 recorded as the primary or first secondary diagnosis on at least two separate healthcare dates during 2011–2022 were classified as meeting the definition of diagnosis-based incident MCI. Requiring the diagnosis on at least two separate dates was intended to reduce potential misclassification from a single provisional or erroneous diagnostic record and to improve the specificity of case ascertainment. However, this operational definition has not been independently validated against standardized clinical or neuropsychological criteria for MCI. Therefore, information on the cognitive domains assessed, diagnostic cut-offs, adjustment for age or education, and preservation of activities of daily living or functional independence was not available, and fulfillment of established clinical MCI criteria could not be independently verified.

### 2.4. Study Variables

Study variables were selected based on previous research and availability in the NHIS customized database and grouped into five domains: sociodemographic, physiological, lifestyle-related, reproductive, and comorbidity factors.

Sociodemographic, physiological, and lifestyle-related factors were assessed using health-screening data from 2009–2010 and, where available for longitudinal-change analyses, corresponding measurements from 2021–2022. Longitudinal changes were evaluated for economic status, employment status, body mass index (BMI), hemoglobin, total cholesterol, smoking, alcohol consumption, and physical activity.

Sociodemographic factors included age, economic status, and employment status. Economic status was based on NHIS insurance premiums and categorized into five groups from low to high. Employment status was determined from employee health insurance subscriber status.

Physiological factors included BMI, hemoglobin, and total cholesterol. BMI was categorized according to the 2022 criteria of the Korean Society for the Study of Obesity, hemoglobin according to the World Health Organization criterion for anemia in women, and total cholesterol according to the 2022 guidelines of the Korean Society of Lipid and Atherosclerosis.

Lifestyle-related factors included smoking, alcohol consumption, and physical activity. Smoking was categorized as current smoking or non-smoking; alcohol consumption as lower- or higher-risk drinking according to NHIS health screening criteria; and physical activity as insufficient or adequate, with adequate activity defined as moderate- or higher-intensity physical activity at least three times per week.

Reproductive factors included age at menarche, age at menopause, hormone replacement therapy, parity, breastfeeding history, and oral contraceptive use obtained from the 2021–2022 cancer-screening questionnaires.

Comorbidity factors included hypertension, diabetes mellitus, osteoporosis, major depressive disorder, sleep disturbance, and hearing loss. These comorbidities were ascertained during the 2011–2022 follow-up period. A comorbidity was considered present when the corresponding KCD diagnosis was recorded as the primary or first secondary diagnosis on at least two separate healthcare dates during 2011–2022. Medication information was considered in the eligibility and exclusion criteria used to construct the baseline cohort in 2009–2010. However, longitudinal medication claims were not included in the customized analytic database available for the 2011–2022 follow-up period. Therefore, comorbidities during follow-up were ascertained using diagnosis code-based operational definitions without incorporating medication claims. Because comorbidities and diagnosis-based incident MCI were ascertained during the same follow-up period, their temporal ordering could not be established for all participants.

Longitudinal-change variables were derived by comparing measurements obtained during the 2009–2010 baseline assessment with those obtained during the 2021–2022 follow-up assessment. For longitudinal-change variables, “no change” indicated that the participant remained in the same category at both assessment periods. For economic status, BMI, and total cholesterol, a decrease or increase indicated movement to a lower or higher predefined category, respectively. Employment status was classified as no change, became unemployed, or became employed. Smoking and alcohol consumption changes reflected transitions between their respective categories. For physical activity, a decrease indicated a change from the higher-activity category to the lower-activity category, whereas an increase indicated a change from the lower-activity category to the higher-activity category. For hemoglobin, changes were expressed explicitly as <12 to ≥12 g/dL or ≥12 to <12 g/dL, with participants remaining in the same hemoglobin category classified as no change.

These two assessment periods were used because they represented the baseline and follow-up health-screening measurements available for defining changes in the study dataset. Because diagnosis-based incident MCI was ascertained throughout 2011–2022, the temporal ordering between meeting the definition of diagnosis-based incident MCI and changes measured at the 2021–2022 assessment could not be established for all participants. Therefore, these longitudinal-change variables were examined as factors associated with diagnosis-based incident MCI rather than as exposures or predictors that necessarily preceded its ascertainment.

Detailed definitions, data sources, categorization criteria, and definitions of longitudinal changes are provided in Supplementary Table S1.

### 2.5. Statistical Analysis

All analyses were performed using SAS Enterprise Guide running on SAS 9.4 (SAS Institute Inc., Cary, NC, USA) within the secure analysis environment of the NHIS Big Data Analysis Center. In accordance with NHIS data-access procedures, the analyses were conducted on authorized computers at the center. Statistical significance was set at *p* < 0.05. Continuous variables were summarized as means and standard deviations, and categorical variables as frequencies and percentages. Characteristics of women with and without diagnosis-based incident MCI were compared using *t*-tests for continuous variables and chi-square tests for categorical variables.

Multivariable logistic regression was used to examine factors associated with diagnosis-based incident MCI. For sociodemographic, physiological, and lifestyle-related factors assessed in both 2009–2010 and 2021–2022, longitudinal-change variables were derived by comparing the two assessment periods. Reproductive variables were obtained from the 2021–2022 cancer-screening questionnaires, and comorbidities were ascertained from diagnostic information recorded during 2011–2022. The model included age, change variables for economic status, employment status, BMI, hemoglobin, total cholesterol, and physical activity, as well as reproductive and comorbidity factors. For BMI, hemoglobin, and total cholesterol, the multivariable models included the derived change variables rather than the corresponding baseline values as separate covariates. Associations were reported as odds ratios (ORs) with 95% confidence intervals (CIs).

For the longitudinal-change variables, between-group differences were initially examined using bivariate analyses, and variables showing statistically significant between-group differences were subsequently included in the multivariable logistic regression model. Changes in smoking and alcohol consumption were not included in the CCA multivariable model because they were not statistically significant in the preliminary bivariate analyses. Consequently, the covariate sets were not identical between the complete-case and analyses of the five separately imputed datasets with respect to smoking and alcohol-related variables, limiting direct comparability of these variables between the two approaches.

Because longitudinal changes were defined by comparing measurements obtained in 2009–2010 and 2021–2022, whereas diagnosis-based incident MCI was ascertained throughout 2011–2022, the second assessment may have occurred after participants met the definition of diagnosis-based incident MCI. In addition, reproductive variables were obtained in 2021–2022, while comorbidities and diagnosis-based incident MCI were ascertained during the same 2011–2022 follow-up period. Therefore, temporal precedence relative to ascertainment of diagnosis-based incident MCI could not be established for all variables. Accordingly, the analyses were interpreted as identifying factors associated with diagnosis-based incident MCI rather than antecedent predictors or causal risk factors.

Results from the CCA were descriptively compared with those from the five separately imputed datasets with respect to the direction, magnitude, and statistical significance of the observed associations.

As an exploratory sensitivity analysis, a broader composite cognitive and neurological outcome was defined to include MCI, dementia, Alzheimer’s disease, cerebrovascular disease, Parkinson’s disease, and other prespecified neurological disorders that were excluded from the primary analytic cohort. Because these conditions are clinically heterogeneous, this composite outcome was not intended to represent a single homogeneous clinical endpoint or to validate the primary diagnosis-based MCI findings. The same covariate set was applied to the broader composite outcome in each of the five imputed datasets, and the results were descriptively examined for the direction, magnitude, and pattern of associations (Supplementary Table S3).

### 2.6. Missing Data Handling

Missingness varied across study variables. Total cholesterol in 2022 had the highest proportion of missing values (56.3%). Missingness in reproductive variables ranged from 23.3% to 39.8%. Variable-specific numbers and proportions of missing observations before missing-data handling are provided in Supplementary Table S2. Missingness stratified by MCI status and direct comparisons between participants with complete and incomplete data were not available in the archived outputs.

For CCA, participants with at least one missing value among variables included in the analysis were excluded. The dataset before complete-case exclusion contained 2,178,195 women, among whom 6.9% met the diagnosis-based incident MCI definition. Imputation was conducted using this pre-complete-case dataset. Five imputed datasets were generated using PROC MI in SAS (NIMPUTE = 5, SEED = 12345). No FCS, MONOTONE, or METHOD option was explicitly specified; therefore, the default Markov chain Monte Carlo (MCMC) procedure based on a multivariate normal model was used. The MCI outcome variable was not included in the imputation model. No ROUND = option was specified in PROC MI. Following imputation, derived variables used in the regression analyses were categorized according to the predefined coding rules applied in this study.

Multivariable logistic regression was performed separately within each of the five imputed datasets. Estimates and variances from the five imputed datasets were not pooled using Rubin’s rules. Instead, results from the five separately imputed datasets were descriptively summarized using observed values or minimum-to-maximum ranges of ORs, 95% CIs, and *p* values across the five datasets and were descriptively compared with the CCA findings. Because the archived aggregate summaries did not permit reliable reconstruction of the exact sample size and number of MCI cases in each individual imputed regression dataset, potentially inaccurate per-imputation counts were not reported.

### 2.7. Ethical Considerations

This study used de-identified customized data provided by the NHIS. The original study received an exemption determination from the Institutional Review Board of Seoul National University (IRB No. E2307/001-011). Access to and use of the NHIS customized database were subsequently reviewed and approved by the NHIS Medical Big Data Review Committee (NHIS-2023-1-813).

### 2.8. Use of Generative Artificial Intelligence

Generative artificial intelligence (ChatGPTGPT-5.6 Sol, OpenAI, San Francisco, CA, USA) was used for English translation and language editing during manuscript preparation. All AI-assisted content was reviewed and verified by the authors, who take full responsibility for the final content of the manuscript.

## 3. Results

### 3.1. Participant Characteristics

After application of the eligibility and disease-related exclusion criteria and removal of duplicate records, 2,178,195 women remained before missing-data handling. For complete-case analysis (CCA), 1,664,316 participants with at least one missing value and 30,695 participants with outlying values were excluded, resulting in a final sample of 483,184 women. Of these, 34,976 (7.2%) met the definition of diagnosis-based incident MCI during 2011–2022.

Baseline characteristics of the complete-case sample are presented in Table 1. Women who met the definition of diagnosis-based incident MCI were older at baseline than those who did not (54.6 ± 6.1 vs. 49.5 ± 6.6 years, *p* < 0.0001) and had higher mean body mass index (BMI; 23.8 ± 2.7 vs. 23.4 ± 2.8 kg/m^2^, *p* < 0.0001) and total cholesterol levels (204.0 ± 36.0 vs. 198.6 ± 35.2 mg/dL, *p* < 0.0001). Adequate physical activity was less frequent among women who met the definition of diagnosis-based incident MCI (71.1% vs. 73.8%, *p* < 0.0001). Current smoking and higher-risk alcohol consumption did not differ significantly between the groups.

### 3.2. Factors Associated with Diagnosis-Based Incident Mild Cognitive Impairment in the Complete-Case Analysis

Multivariable logistic regression results for the complete-case sample are presented in Table 2 and Table 3. Associations involving longitudinal-change variables are presented in Table 2. Among sociodemographic factors, each one-year increase in age was associated with higher odds of diagnosis-based incident MCI (OR 1.096, 95% CI 1.094–1.099, *p* < 0.0001). Compared with no change in employment status, becoming unemployed was associated with lower odds (OR 0.91, 95% CI 0.872–0.939), whereas becoming employed was associated with higher odds of diagnosis-based incident MCI (OR 1.04, 95% CI 1.007–1.083). An increase in BMI category (OR 1.04, 95% CI 1.007–1.064, *p* = 0.02) and a change from insufficient to adequate physical activity (OR 1.07, 95% CI 1.044–1.102, *p* < 0.0001) were also associated with higher odds of diagnosis-based incident MCI (Table 2).

Associations involving reproductive and comorbidity factors are presented in Table 3. Among reproductive factors, age at menarche (OR 1.04, 95% CI 1.031–1.045), hormone replacement therapy (OR 1.36, 95% CI 1.325–1.401), and oral contraceptive use (OR 1.05, 95% CI 1.020–1.086) were significantly associated with diagnosis-based incident MCI, whereas age at menopause, parity, and breastfeeding history were not. Among comorbidity factors, diabetes mellitus, osteoporosis, major depressive disorder, sleep disturbance, and hearing loss were significantly associated with higher odds of diagnosis-based incident MCI, with ORs ranging from 1.16 for diabetes mellitus to 1.73 for major depressive disorder. The largest associations among the examined comorbidities were observed for major depressive disorder (OR 1.73), sleep disturbance (OR 1.53), and osteoporosis (OR 1.52). In contrast, several statistically significant associations in other domains had effect estimates close to the null, including changes in employment status, increased BMI category, age at menarche, and oral contraceptive use. Hypertension was not statistically significant (OR 1.01, 95% CI 0.981–1.030, *p* = 0.67) (Table 3).

### 3.3. Descriptive Comparison of Factors Associated with Diagnosis-Based Incident Mild Cognitive Impairment Between the Complete-Case Analysis and Five Separately Imputed Datasets

Results from the CCA and the five separately imputed datasets are descriptively compared in Table 4. Age, changes in employment status, an increase in BMI category, a change from insufficient to adequate physical activity, age at menarche, hormone replacement therapy, oral contraceptive use, diabetes mellitus, osteoporosis, major depressive disorder, sleep disturbance, and hearing loss showed similar patterns of association with diagnosis-based incident MCI in the CCA and across the five imputed datasets.

Differences in the observed patterns of association were noted for several factors. A decrease in economic status, a decrease in BMI category, a change in hemoglobin from ≥12 to <12 g/dL, an increase in total cholesterol category, a change from adequate to insufficient physical activity, parity, breastfeeding history, and hypertension were not statistically significant in the CCA but showed statistically significant associations with diagnosis-based incident MCI across the five separately imputed datasets. Smoking and changes in alcohol consumption were not included in the CCA multivariable model because they did not meet the preliminary bivariate screening criterion. Although statistically significant associations with these variables were observed across the five separately imputed datasets, these findings were not considered directly comparable with the CCA results because the covariate sets differed between the two approaches. Age at menopause was not significantly associated with diagnosis-based incident MCI in either the CCA or across the five separately imputed datasets.

Overall, several patterns of association were similar in the CCA and across the five separately imputed datasets, whereas differences were observed for some variables. Direct comparison was limited to variables that were evaluated in both approaches. Because estimates from the five imputed datasets were not pooled using Rubin’s rules, these comparisons are descriptive and should not be interpreted as conventional pooled MI inference.

As an additional sensitivity analysis to examine the influence of the disease-related exclusions in the primary analytic cohort, MCI, dementia, Alzheimer’s disease, cerebrovascular disease, Parkinson’s disease, and other prespecified neurological disorders were analyzed together as a broader composite cognitive and neurological outcome. The overall direction and magnitude of several major associations were generally similar to those observed in the primary analyses of the five separately imputed datasets (Supplementary Table S3). In particular, the associations for diabetes mellitus, osteoporosis, major depressive disorder, sleep disturbance, and hearing loss remained similar in direction and magnitude across the five imputed datasets. Associations with age at menarche, hormone replacement therapy, breastfeeding history, and oral contraceptive use were also generally similar to those observed in the primary analyses of the five separately imputed datasets, whereas age at menopause and parity were not statistically significant in the sensitivity analysis.

## 4. Discussion

This study examined factors associated with diagnosis-based incident mild cognitive impairment (MCI) among Korean women who entered the cohort during midlife using 14 years of National Health Insurance Service (NHIS) data and descriptively compared findings from complete-case analysis (CCA) with results from five separately imputed datasets. Several sociodemographic, physiological, lifestyle-related, reproductive, and comorbidity-related factors showed similar associations in the CCA and across the five separately imputed datasets, whereas additional statistically significant associations were observed in the five separately imputed datasets. Associations were observed across multiple health domains, although the specific factors identified differed to some extent between the CCA and results from the five separately imputed datasets.

Among the comorbidities examined, diabetes mellitus, osteoporosis, major depressive disorder, sleep disturbance, and hearing loss showed statistically significant associations with diagnosis-based incident MCI in the CCA and across the five separately imputed datasets. These findings are consistent with previous studies linking chronic conditions, depression, sleep problems, and hearing impairment with cognitive impairment or dementia [15,16,17,18,19]. Notably, major depressive disorder showed one of the stronger comorbidity-related associations in the present study, with approximately 1.7-fold higher odds of diagnosis-based incident MCI. This magnitude is broadly comparable to that reported in the Women’s Health Initiative Memory Study, in which depressive disorder was associated with an adjusted HR of 1.98 (95% CI, 1.33–2.94) for incident MCI among older postmenopausal women [20]. Although the effect estimates are not directly comparable because of differences in participant age, outcome and exposure ascertainment, follow-up, and effect measures (OR versus HR), the findings suggest a broadly similar magnitude of association. However, because comorbidities and diagnosis-based incident MCI were ascertained during the same 2011–2022 follow-up period, the temporal direction of these associations could not be established. Hypertension was statistically significant only in results from the five separately imputed datasets, indicating that some comorbidity-related associations differed between the CCA and results from the five separately imputed datasets. Given the large sample size, statistical significance should be interpreted in conjunction with effect size. Several statistically significant associations had ORs close to 1.0 and should therefore not be assumed to represent clinically important associations, whereas relatively larger associations were observed for major depressive disorder (OR 1.73), sleep disturbance (OR 1.53), and osteoporosis (OR 1.52).

Reproductive characteristics also showed associations with diagnosis-based incident MCI. Age at menarche, hormone replacement therapy, and oral contraceptive use showed statistically significant associations with diagnosis-based incident MCI in the CCA and across the five separately imputed datasets, whereas parity and breastfeeding history were statistically significant only in results from the five separately imputed datasets. Age at menopause was not significantly associated with diagnosis-based incident MCI in either approach. Previous studies have suggested that reproductive history and endogenous and exogenous hormone exposure may be related to cognitive health in women, although the direction and magnitude of these associations have varied [21,22,23,24]. Reproductive characteristics may also reflect broader biological, socioeconomic, and health-related experiences accumulated across the life course. Therefore, the present findings should not be interpreted as evidence that hormone replacement therapy or oral contraceptive use causes cognitive impairment. Rather, these findings highlight the need for further studies to clarify how reproductive history relates to cognitive health across midlife.

The menopausal transition also warrants consideration when interpreting MCI identified in this midlife female cohort. Longitudinal evidence suggests that the menopausal transition may be accompanied by modest changes in cognitive performance, including memory and processing speed, although these changes generally remain within the normal range and do not necessarily indicate pathological cognitive impairment [6,25]. In the present study, age at menopause was included as a reproductive characteristic; however, menopausal status at the time of MCI diagnosis, time since menopause, and whether MCI was diagnosed during the menopausal transition or after menopause were not evaluated. Therefore, transient cognitive changes occurring around the menopausal transition could not be distinguished from persistent cognitive impairment among women with diagnosis-based MCI, and this possibility should be considered when interpreting the findings.

The associations observed across sociodemographic, physiological, lifestyle-related, reproductive, and comorbidity domains indicate that reproductive characteristics should be considered within the broader health context of midlife women rather than in isolation. In particular, the concurrent associations of reproductive characteristics with physical, psychological, sleep-related, and sensory health factors support further investigation of cognitive health using an integrated, multidomain approach. However, because temporal precedence was not established for all factors, these associations should not be interpreted as causal relationships.

The diagnosis-based identification of MCI in the present study should be interpreted in relation to established clinical criteria. Petersen and colleagues initially characterized MCI as a clinical state distinct from normal aging and dementia, and subsequent criteria have emphasized objective impairment in one or more cognitive domains with relatively preserved functional independence [1,26]. Because the NHIS healthcare database did not provide the neuropsychological or functional assessment data underlying individual diagnoses, correspondence between the diagnosis-based MCI definition used in this study and clinically adjudicated MCI according to these established criteria could not be determined.

The exclusion of participants with dementia and other prespecified neurological disorders during the 2011–2022 follow-up period also warrants consideration when interpreting the MCI outcome. Because these exclusions were applied across the entire follow-up period without distinguishing whether the qualifying diagnosis occurred before or after ascertainment of diagnosis-based incident MCI, some individuals who subsequently developed dementia after meeting the definition of diagnosis-based incident MCI may have been excluded from the analytic cohort. Consequently, the MCI cases retained in the primary analysis may represent a selected subset of diagnosis-based MCI rather than the full spectrum of MCI clinical trajectories. In a sensitivity analysis incorporating MCI and the excluded dementia, Alzheimer’s disease, cerebrovascular disease, Parkinson’s disease, and other neurological disorders into a broader composite cognitive and neurological outcome, the overall direction and magnitude of several major associations were generally similar to those observed in the primary analyses of the five separately imputed datasets, although age at menopause and parity were not consistently associated with the broader outcome. These exploratory findings indicated broadly similar patterns for several associations under the broader outcome definition; however, they should not be interpreted as confirmation of the primary diagnosis-based incident MCI findings. The sensitivity analysis also does not establish the temporal sequence of MCI-to-dementia progression or replace formal time-to-event or competing-risk analyses. Future longitudinal studies that retain individuals after MCI identification and explicitly examine progression from MCI to dementia are needed.

The longitudinal-change findings require cautious interpretation because the temporal ordering of these changes relative to ascertainment of diagnosis-based incident MCI could not be established. A change from insufficient to adequate physical activity was associated with higher odds of diagnosis-based incident MCI, whereas becoming unemployed was associated with lower odds and becoming employed with higher odds. These counterintuitive associations should not be interpreted as evidence that increasing physical activity is harmful or that becoming unemployed is protective against MCI. Changes in these variables were determined by comparing assessments conducted in 2009–2010 and 2021–2022, whereas diagnosis-based incident MCI was ascertained throughout 2011–2022. Thus, for some participants, meeting the definition of diagnosis-based incident MCI may have preceded the second assessment, and reverse causality cannot be excluded. Emerging cognitive impairment or related health changes may themselves have contributed to subsequent changes in physical activity, employment, BMI, or other health behaviors. Employment transitions may also reflect retirement, health-related changes in work participation, caregiving responsibilities, or socioeconomic circumstances that were not distinguishable in the available data. Temporal precedence was also not established for all reproductive and comorbidity factors because reproductive variables were obtained from the 2021–2022 screening data and comorbidities were ascertained during 2011–2022. Therefore, the observed associations should be interpreted as factors associated with diagnosis-based incident MCI rather than as temporally established predictors, risk factors, or protective factors.

An important methodological observation was that the patterns of factors associated with diagnosis-based incident MCI differed to some extent between the CCA and results from the five separately imputed datasets. Several socioeconomic, physiological, lifestyle-related, reproductive, and comorbidity-related factors showed statistically significant associations across the five imputed datasets but not in the CCA. Smoking and changes in alcohol consumption were not included in the CCA multivariable model because they did not meet the preliminary bivariate screening criterion. Although statistically significant associations with these variables were observed across the five imputed datasets, these findings were not considered directly comparable with the CCA results because the covariate sets differed between the two approaches. Therefore, differences involving smoking and alcohol-related variables cannot be attributed specifically to the missing-data handling approach.

CCA excluded a substantial proportion of participants because of missing values, whereas the five imputed datasets were generated from the dataset before listwise deletion. The extent of missingness varied considerably across variables, with particularly high missingness for total cholesterol and reproductive characteristics. Previous methodological research has shown that CCA can reduce the analytic sample and may yield biased estimates when complete cases differ systematically from participants with incomplete data, whereas MI can retain observed information that would otherwise be excluded [10,11,12]. However, the validity of MI depends on the assumptions underlying the imputation model. In the present study, MI was implemented under a missing-at-random (MAR) assumption; however, the true missing-data mechanism could not be empirically determined, and missing not at random (MNAR) cannot be excluded.

Importantly, the five imputed datasets were analyzed separately, and estimates and variances were not pooled using Rubin’s rules. Consequently, the confidence intervals reported from the five separately imputed datasets reflect within-imputation uncertainty but do not incorporate between-imputation variability. The observed differences between the CCA and results from the five separately imputed datasets should therefore be interpreted descriptively and should not be considered evidence that MI produced more accurate, precise, or less biased estimates than CCA. Rather, the findings indicate that the observed pattern of associations may vary according to the approach used to handle missing data, while differences in model specification also limit direct comparison for some variables. For research using routinely collected healthcare data, transparent reporting of the extent and pattern of missingness, assumptions regarding the missing-data mechanism, and methods used to address missing data is important for appropriately interpreting the resulting evidence.

In clinical practice, middle-aged women are routinely assessed for chronic conditions, psychological health, sleep, health behaviors, and reproductive history in health screening, primary care, outpatient, and community settings. The associations observed in this study suggest that cognitive health could be considered within this broader health context rather than only after cognitive symptoms become apparent. More broadly, integrating information across multiple health domains may provide a more comprehensive basis for future data-driven assessment than considering individual factors in isolation. Although the present study differs substantially from predictive modeling or pharmacovigilance applications in population, outcome, and clinical purpose, similar methodological principles apply to the integration of heterogeneous clinical, behavioral, and contextual information.

However, the identified factors should not be regarded as independent causal predictors or as components of a validated clinical prediction model. Translation of multidomain observational findings into individualized risk assessment or clinical decision-support tools would require prospective and external validation, assessment of transportability across populations and healthcare settings, clinician interpretation, and consideration of implementation barriers. Accordingly, the present findings should be viewed as hypothesis-generating evidence for future multidomain assessment rather than as a validated basis for clinical decision support.

Several limitations should be considered when interpreting the findings. First, MCI and comorbidities were ascertained from KCD-based diagnostic information in the NHIS healthcare database rather than standardized clinical or neuropsychological assessments. Although requiring F06.7 on at least two separate healthcare dates was intended to improve diagnostic specificity, this approach may have reduced sensitivity by excluding individuals with only a single F06.7 diagnosis. If such outcome misclassification was largely nondifferential with respect to the examined factors, it could have attenuated some associations toward the null. However, differential misclassification related to healthcare utilization or comorbidity cannot be excluded; therefore, the direction and magnitude of potential bias cannot be determined from the available data. Because comorbidities and diagnosis-based incident MCI were ascertained during the same 2011–2022 follow-up period, their temporal ordering could not be established, limiting their interpretation as antecedent risk factors. Menopausal status at the time of diagnosis-based incident MCI ascertainment and time since menopause were also unavailable; therefore, transient cognitive changes occurring around the menopausal transition could not be distinguished from persistent cognitive impairment among women with diagnosis-based MCI. Reproductive history was also self-reported in the 2021–2022 cancer-screening questionnaires, which may have introduced recall error, particularly among women who had already met the definition of diagnosis-based incident MCI. Ascertainment was limited to the primary and first secondary diagnosis codes, which may have resulted in under-ascertainment or differential misclassification of MCI and comorbidities. The exclusion of participants who died during 2009–2022 or met prespecified criteria for dementia, Alzheimer’s disease, cerebrovascular disease, Parkinson’s disease, or other neurological disorders during the 2011–2022 follow-up period may also have introduced selection bias because some exclusions occurred after cohort entry. Although the sensitivity analysis using a broader composite cognitive and neurological outcome showed generally similar patterns of association, it did not address MCI-to-dementia progression or replace formal time-to-event or competing-risk analyses. The logistic regression approach also did not account for time to MCI diagnosis, censoring, or the competing risk of death.

Second, medication information was incorporated into the eligibility and exclusion criteria used to construct the baseline cohort but was unavailable for comorbidity ascertainment during the 2011–2022 follow-up period. Consequently, pharmacologically treated conditions, particularly hypertension and diabetes, may have been incompletely ascertained compared with algorithms incorporating both diagnostic and prescription information.

Third, missingness varied substantially across study variables, and MI was conducted under a missing-at-random (MAR) assumption that could not be empirically verified; missing not at random (MNAR) therefore cannot be excluded. Only approximately 22% of the pre-complete-case cohort was retained in the CCA, which may have introduced selection related to missingness. Although variable-specific missingness was reported for the pre-complete-case cohort, missingness could not be stratified by MCI status and complete versus incomplete participants could not be directly compared from the archived outputs. The default MCMC approach assumed multivariate normality and may not have been optimal for some binary and categorical variables. In addition, the MCI outcome was not included in the imputation model. Only five imputed datasets were generated, and the estimates were analyzed separately rather than pooled using Rubin’s rules. Therefore, between-imputation variability was not incorporated into the reported confidence intervals, and results from the five separately imputed datasets should be interpreted descriptively rather than as conventional pooled multiple-imputation estimates. Smoking- and alcohol-related variables were also excluded from the CCA multivariable model after preliminary bivariate screening, resulting in nonidentical covariate sets between the CCA and analyses of the five separately imputed datasets. These differences limit direct comparison between the two approaches and should not be interpreted as evidence that either approach was superior.

Finally, although the large national healthcare database provided broad population coverage, the cohort was restricted to Korean women who participated in the specified health screening programs. Therefore, caution is warranted when generalizing the findings to women who did not participate in health screening, populations in other healthcare systems, or men.

## 5. Conclusions

This study identified factors associated with diagnosis-based incident MCI among Korean women who entered the cohort during midlife. The pattern of observed associations differed between the complete-case analysis and the five separately imputed datasets analyzed without Rubin’s-rule pooling; however, these differences should be interpreted descriptively because the analytic approaches also differed in covariate specification and outlier handling. Given that some covariates and diagnosis-based incident MCI were ascertained over overlapping periods, temporal ordering could not be established for all associations. These findings underscore the importance of transparent reporting of missingness, missing-data handling procedures, analytic differences, and temporal limitations when interpreting associations derived from longitudinal healthcare database studies.

## Figures and Tables

**Table 1:** Baseline characteristics of the complete-case sample according to diagnosis-based incident mild cognitive impairment status.

Characteristics	Categories	MCI (n = 34,976)	Non-MCI (n = 448,208)	*p* Value
Sociodemographic factors				
Age, years	Mean ± SD	54.6 ± 6.1	49.5 ± 6.6	<0.001
Economic status				<0.001
	Low	7259 (20.8)	96,080 (21.4)	
	Lower-middle	6073 (17.4)	83,043 (18.5)	
	Middle	6192 (17.7)	75,570 (16.9)	
	Upper-middle	7447 (21.3)	86,682 (19.3)	
	High	8005 (22.9)	106,833 (23.8)	
Employment status				<0.001
	Not employed	26,806 (76.6)	305,715 (68.2)	
	Employed	8170 (23.4)	142,493 (31.8)	
Physiological factors				
BMI, kg/m^2^	Mean ± SD	23.80 ± 2.70	23.40 ± 2.80	<0.001
BMI category				<0.001
	<18.5	486 (1.4)	10,031 (2.2)	
	18.5–<23	13,487 (38.6)	205,001 (45.7)	
	23–<25	9926 (28.4)	114,421 (25.5)	
	≥25	11,077 (31.7)	118,755 (26.5)	
Hemoglobin, g/dL	Mean ± SD	12.90 ± 1.00	12.80 ± 1.10	<0.001
Hemoglobin category				<0.001
	<12	5133 (14.7)	79,607 (17.8)	
	≥12	29,843 (85.3)	368,601 (82.2)	
Total cholesterol, mg/dL	Mean ± SD	204.00 ± 36.00	198.60 ± 35.20	<0.001
Total cholesterol category				<0.001
	<200	16,670 (47.7)	242,662 (54.1)	
	200–239	12,762 (36.5)	149,091 (33.3)	
	≥240	5544 (15.9)	56,455 (12.6)	
Health behavior factors				
Smoking status	Non-smoker ^(a)^	34,167 (97.7)	437,462 (97.6)	0.320
	Current smoker	809 (2.3)	10,746 (2.4)	
Alcohol consumption	Lower risk	32,565 (93.1)	417,444 (93.1)	0.830
	Higher risk	2411 (6.9)	30,764 (6.9)	
Physical activity	Insufficient	10,121 (28.9)	117,599 (26.2)	<0.001
	Adequate	24,855 (71.1)	330,609 (73.8)	

Note. Values are presented as mean ± standard deviation or n (%). Baseline characteristics were assessed using data from 2009–2010. The MCI group comprised women who met the definition of diagnosis-based incident MCI during 2011–2022; women with MCI at baseline were excluded. BMI, body mass index; MCI, mild cognitive impairment; SD, standard deviation. ^(a)^ The non-smoking category included former smokers.

**Table 2:** Associations of longitudinal changes in sociodemographic, physiological, and lifestyle-related factors with diagnosis-based incident mild cognitive impairment in the complete-case analysis.

Factors	Variables	Categories	OR	95% CI	*p* Value
Sociodemographic	Age, years	Continuous	1.096	1.094–1.099	<0.001
	Change in economic status	No change	Ref.		
		Decreased	1.02	0.991–1.053	0.350
		Increased	1.02	0.989–1.046	0.600
	Change in employment	No change	Ref.		
		Became unemployed	0.91	0.872–0.939	<0.001
		Became employed	1.04	1.007–1.083	—
Physiological	Change in BMI group	No change	Ref.		
		Decreased	1.00	0.966–1.035	0.330
		Increased	1.04	1.007–1.064	0.020
	Change in hemoglobin level	No change	Ref.		
		<12 → ≥12 g/dL	1.00	0.969–1.039	0.170
		≥12 → <12 g/dL	0.95	0.901–1.000	0.050
	Change in total cholesterol level	No change	Ref.		
		Decreased	1.01	0.980–1.034	0.360
		Increased	0.99	0.962–1.018	0.300
Lifestyle-related	Change in physical activity	No change	Ref.		
		Adequate → insufficient	1.01	0.968–1.047	0.160
		Insufficient → adequate	1.07	1.044–1.102	<0.001

Note. The complete-case analysis included 483,184 women after exclusion of participants with missing data and statistical outliers according to the predefined analytic procedure. Changes in economic status, employment status, BMI, hemoglobin, total cholesterol, and physical activity were determined by comparing individual-level data from 2009–2010 with corresponding data from 2021–2022. These change variables represent transitions between predefined categories. ORs and 95% CIs were estimated using multivariable logistic regression. Changes in smoking and alcohol consumption were not included in the complete-case multivariable model because they were not statistically significant in the preliminary bivariate analyses. Because the 2021–2022 assessment period overlapped with the follow-up period for diagnosis-based incident MCI (2011–2022), these associations should not be interpreted as temporally established predictors or causal effects. The variables presented in Table 2 and Table 3 were included in the same multivariable logistic regression model. For the “became employed” category, the archived *p* value was not fully concordant with the reported OR and 95% CI and could not be reliably verified from the available output; therefore, the *p* value was not reported (—). BMI, body mass index; CI, confidence interval; MCI, mild cognitive impairment; OR, odds ratio; Ref., reference.

**Table 3:** Reproductive and comorbidity factors associated with diagnosis-based incident mild cognitive impairment in the complete-case analysis.

Factors	Variables	Categories	OR	95% CI	*p* Value
Reproductive factors					
	Age at menarche, years	Continuous	1.04	1.031–1.045	<0.001
	Age at menopause, years	Continuous	1.00	0.998–1.004	0.600
	Hormone replacement therapy	No	Ref.		
		Yes	1.36	1.325–1.401	<0.001
	Parity	No	Ref.		
		Yes	1.09	0.965–1.229	0.170
	Breastfeeding history	No	Ref.		
		Yes	1.03	0.988–1.080	0.150
	Oral contraceptive use	No	Ref.		
		Yes	1.05	1.020–1.086	0.0014
Comorbidity-related factors					
	Hypertension	No	Ref.		
		Yes	1.01	0.981–1.030	0.670
	Diabetes mellitus	No	Ref.		
		Yes	1.16	1.131–1.187	<0.001
	Osteoporosis	No	Ref.		
		Yes	1.52	1.484–1.561	<0.001
	Major depressive disorder	No	Ref.		
		Yes	1.73	1.686–1.777	<0.001
	Sleep disturbance	No	Ref.		
		Yes	1.53	1.491–1.566	<0.001
	Hearing loss	No	Ref.		
		Yes	1.30	1.261–1.330	<0.001

Note. The complete-case analysis included 483,184 women after exclusion of participants with missing data and statistical outliers according to the predefined analytic procedure. ORs and 95% CIs were estimated using multivariable logistic regression. Comorbidities were defined as the relevant KCD code recorded as the primary or first secondary diagnosis on at least two separate healthcare dates during follow-up. The variables presented in Table 2 and Table 3 were included in the same multivariable logistic regression model. CI, confidence interval; KCD, Korean Standard Classification of Diseases; MCI, mild cognitive impairment; OR, odds ratio; Ref., reference.

**Table 4:** Descriptive comparison of complete-case estimates and results from five separately imputed datasets for diagnosis-based incident mild cognitive impairment.

Factor	Predictor/Category	Complete-Case Analysis		Five Separately Imputed Datasets (Descriptive)	
		OR (95% CI)	*p* Value	OR (95% CI)	*p* Value
Sociodemographic factors	Age, per year	1.096 (1.094–1.099)	<0.001	1.10 (1.099–1.100)	<0.001
	Change in economic status				
	No change	Ref.		Ref.	
	Decreased	1.02 (0.991–1.053)	0.350	1.03 (1.025–1.040)	<0.001
	Increased	1.02 (0.989–1.046)	0.600	1.00 (0.992–1.006)	-
	Change in employment status				
	No change	Ref.		Ref.	
	Became unemployed	0.91 (0.872–0.939)	<0.001	0.98 (0.974–0.990)	<0.001
	Became employed	1.04 (1.007–1.083)	-	1.09 (1.081–1.099)	<0.001
Physiological factors	Change in BMI group				
	No change	Ref.		Ref.	
	Decreased	1.00 (0.966–1.035)	0.330	0.91 (0.904–0.917)	<0.001
	Increased	1.04 (1.007–1.064)	0.020	1.06 (1.048–1.062)	<0.001
	Change in hemoglobin level				
	No change	Ref.		Ref.	
	<12 → ≥12 g/dL	1.00 (0.969–1.039)	0.170	1.00 (0.993–1.010)	-
	≥12 → <12 g/dL	0.95 (0.901–1.000)	0.050	0.88 (0.876–0.890)	<0.001
	Change in total cholesterol level				
	No change	Ref.		Ref.	
	Decreased	1.01 (0.980–1.034)	0.360	1.00 (0.998–1.009)	-
	Increased	0.99 (0.962–1.018)	0.300	0.98 (0.975–0.991)	<0.001
Lifestyle-related factors	Change in smoking				
	No change	Ref.		Ref.	
	Decreased ^(a)^	—	—	1.08 (1.061–1.096)	<0.001
	Change in drinking				
	No change	Ref.		Ref.	
	Decreased ^(a)^	—	—	1.01 (0.999–1.024)	0.004–0.006
	Increased ^(a)^	—	—	0.99 (0.978–0.999)	<0.001–0.002
	Change in physical activity				
	No change	Ref.		Ref.	
	Adequate → insufficient	1.01 (0.968–1.047)	0.160	1.02 (1.005–1.024)	<0.001
	Insufficient → adequate	1.07 (1.044–1.102)	<0.001	1.07 (1.062–1.074)	<0.001
Reproductive factors	Age at menarche, per year	1.04 (1.031–1.045)	<0.001	1.03 (1.029–1.032)	<0.001
	Age at menopause, per year	1.00 (0.998–1.004)	0.600	1.00 (0.999–1.000)	0.059–0.075
	Hormone replacement therapy				
	No	Ref.		Ref.	
	Yes	1.36 (1.325–1.401)	<0.001	1.24 (1.229–1.250)	<0.001
	Parity				
	No	Ref.		Ref.	
	Yes	1.09 (0.965–1.229)	0.170	1.02–1.03 (1.005–1.047)	<0.001–0.007
	Breastfeeding history				
	No	Ref.		Ref.	
	Yes	1.03 (0.988–1.080)	0.150	1.05–1.06 (1.041–1.071)	<0.001
	Oral contraceptive use				
	No	Ref.		Ref.	
	Yes	1.05 (1.020–1.086)	<0.01	1.06–1.07 (1.054–1.075)	<0.001
Comorbidity-related factors	Hypertension				
	No	Ref.		Ref.	
	Yes	1.01 (0.981–1.030)	0.670	1.02 (1.015–1.026)	<0.001
	Diabetes mellitus				
	No	Ref.		Ref.	
	Yes	1.16 (1.131–1.187)	<0.001	1.17 (1.162–1.175)	<0.001
	Osteoporosis				
	No	Ref.		Ref.	
	Yes	1.52 (1.484–1.561)	<0.001	1.56 (1.554–1.572)	<0.001
	Major depressive disorder				
	No	Ref.		Ref.	
	Yes	1.73 (1.686–1.777)	<0.001	1.75 (1.741–1.761)	<0.001
	Sleep disturbance				
	No	Ref.		Ref.	
	Yes	1.53 (1.491–1.566)	<0.001	1.55 (1.539–1.556)	<0.001
	Hearing loss				
	No	Ref.		Ref.	
	Yes	1.30 (1.261–1.330)	<0.001	1.32 (1.310–1.326)	<0.001

Note. The complete-case estimates correspond to the multivariable logistic regression results presented in Table 2 and Table 3. Five imputed datasets were generated using PROC MI in SAS and analyzed separately. Results from the five separately imputed datasets are presented descriptively as observed values or minimum-to-maximum ranges of ORs, 95% CIs, and *p* values across the five datasets. Estimates and variances were not pooled using Rubin’s rules; therefore, the reported confidence intervals reflect within-imputation uncertainty only and should not be interpreted as pooled multiple-imputation confidence intervals. An em dash (—) in the complete-case analysis indicates that the variable was not included in the multivariable model. For estimates for which the archived *p* values were not fully concordant with the corresponding ORs and 95% CIs and could not be reliably verified from the available regression outputs, the *p* values are not reported (—). ^(a)^ Changes in smoking and alcohol consumption were excluded from the complete-case model because they were not statistically significant in the preliminary bivariate analyses. Because the complete-case analysis and analyses of the five separately imputed datasets did not use identical covariate sets and differed in data-processing procedures, differences between the two approaches should be interpreted descriptively rather than as evidence of the superiority of either approach. CI, confidence interval; MCI, mild cognitive impairment; OR, odds ratio; Ref., reference.

## Data Availability

The data analyzed in this study were obtained from the National Health Insurance Service (NHIS) of Korea under a data-use agreement and are not publicly available because of restrictions imposed by the NHIS. The authors are not permitted to share individual-level data. Researchers who meet the criteria for access may apply directly to the NHIS for access to the customized database in accordance with NHIS data-access procedures.

## Supplementary Materials

The following supporting information can be downloaded at https://www.mdpi.com/article/10.3390/healthcare14213701/s1. Table S1: Definitions, measurement, and categorization of study variables; Table S2: Missing values in the dataset before missing-data handling; Table S3: Sensitivity analysis of factors associated with a broader composite cognitive and neurological outcome across five imputed datasets.
