# Supplementary material for: Factors Associated with Diagnosis-Based Incident Mild Cognitive Impairment in Middle-Aged Women: A 14-Year National Cohort Study
Source: Healthcare (Basel). 2026 Nov 1;14(21):3701. doi: 10.3390/healthcare14213701 (PMC13762733; doi:10.3390/healthcare14213701)
Supplement: Supplementary file 1 [file healthcare-14-03701-s001.zip › healthcare-4610686-supplementary.pdf]

Supplementary Table S1. Definitions, measurement, and categorization of study variables.

Domain	Variable	Definition/Measurement	Unit/Code	Categorization
**Outcome**	Incident mild cognitive impairment (MCI)	KCD-8 code F06.7 recorded in the primary diagnosis or first secondary diagnosis field on at least two separate healthcare dates	KCD-8: F06.7	Yes/No; the first year meeting the case definition was considered the year of occurrence
**Sociodemographic**	Age	Age at the baseline health screening during 2009–2010	years	Continuous
	Economic status	Defined using NHIS insurance premium level as a proxy for income. Medical Aid beneficiaries were assigned to level 0, and insured participants were classified into levels 1–20, with higher levels indicating higher economic status.	NHIS insurance premium level	Low (0–4)/Lower-middle (5–8)/Middle (9–12)/Upper-middle (13–16)/High (17–20)
	Change in economic status	Change in economic status category between the 2009–2010 and 2021–2022 assessment periods	—	No change/Decrease/Increase
	Employment status	Defined according to workplace-based health insurance subscriber status	—	Not employed/Employed
	Change in employment status	Change in employment status between the 2009–2010 and 2021–2022 assessment periods	—	No change/Became unemployed/Became employed
**Physiological**	Body mass index (BMI)	Body mass index measured at the health screening and categorized according to the 2022 Korean Society for the Study of Obesity criteria	kg/m²	Underweight (<18.5)/Normal weight (18.5–<23)/Overweight (23–<25)/Obesity (≥25)
	Change in BMI	Change in BMI category between the 2009–2010 and 2021–2022 assessment periods	—	No change/Decrease/Increase
	Hemoglobin	Hemoglobin measured at the health screening and categorized according to the World Health Organization criterion for anemia in women	g/dL	Anemia (<12)/Normal (≥12)
	Change in hemoglobin	Change in hemoglobin category between the 2009–2010 and 2021–2022 assessment periods	—	No change/Decrease (normal → anemia)/Increase (anemia → normal)
	Total cholesterol	Total cholesterol measured at the health screening and categorized according to the 2022 Korean Society of Lipid and Atherosclerosis guideline criteria	mg/dL	Desirable (<200)/Borderline high (200–239)/High (≥240)
	Change in total cholesterol	Change in total cholesterol category between the 2009–2010 and 2021–2022 assessment periods	—	No change/Decrease/Increase
**Lifestyle-related**	Smoking	Smoking status assessed using the general health screening questionnaire	—	Non-smoker (including former smokers)/Current smoker
	Change in smoking	Change in category between the 2009–2010 and 2021–2022 assessment periods	—	No change/Decrease (current smoker → non-smoker)/Increase (non-smoker → current smoker)

Domain	Variable	Definition/Measurement	Unit/Code	Categorization
	Alcohol consumption	Alcohol consumption status based on the physician-interpreted health screening result according to Article 7 of the NHIS Health Examination Operational Rules	—	Lower risk (non-drinking or social drinking)/Higher risk (binge or heavy drinking)
	Change in alcohol consumption	Change in alcohol consumption category between the 2009–2010 and 2021–2022 assessment periods	—	No change/Decrease (higher risk → lower risk)/Increase (lower risk → higher risk)
	Physical activity	Physical activity assessed using the general health screening questionnaire	—	Insufficient (<3 sessions/week of moderate-or-higher intensity activity)/Adequate (≥3 sessions/week of moderate-or-higher intensity activity)
	Change in physical activity	Change in physical activity category between the 2009–2010 and 2021–2022 assessment periods	—	No change/Decrease (adequate → insufficient)/Increase (insufficient → adequate)
**Reproductive**	Age at menarche	Self-reported in cancer screening questionnaire	years	Continuous
	Age at menopause	Self-reported in cancer screening questionnaire	years	Continuous
	Hormone replacement therapy	History of HRT use	Yes/No	Yes/No
	Parity	History of childbirth	Yes/No	Yes/No
	Breastfeeding	History of breastfeeding	Yes/No	Yes/No
	Oral contraceptive use	History of oral contraceptive use	Yes/No	Yes/No
**Comorbidity**	Hypertension	KCD-8 codes I10–I15 recorded on at least two separate healthcare dates	KCD-8: I10–I15	Yes/No
	Diabetes mellitus	KCD-8 codes E10–E14 recorded on at least two separate healthcare dates	KCD-8: E10–E14	Yes/No
	Osteoporosis	KCD-8 code M81 recorded on at least two separate healthcare dates	KCD-8: M81	Yes/No
	Major depressive disorder	KCD-8 codes F32–F33 recorded on at least two separate healthcare dates	KCD-8: F32, F33	Yes/No
	Sleep disturbance	KCD-8 codes G47 or F51 recorded on at least two separate healthcare dates	KCD-8: G47, F51	Yes/No
	Hearing loss	KCD-8 codes H90–H91 recorded on at least two separate healthcare dates	KCD-8: H90, H91	Yes/No

Supplementary Table S2. Missing values in the dataset before missing-data handling (N = 2,178,195).

Variable	Available, n	Missing, n (%)
Economic status, 2009	2,159,743	18,452 (0.8)
Economic status, 2022	2,155,003	23,192 (1.1)
BMI, 2009	2,177,252	943 (0.0)
BMI, 2022	1,808,720	369,475 (17.0)
Hemoglobin, 2009	2,177,499	696 (0.0)
Hemoglobin, 2022	1,799,629	378,566 (17.4)
Total cholesterol, 2009	2,177,511	684 (0.0)
Total cholesterol, 2022	952,584	1,225,611 (56.3)
Smoking, 2009	2,166,144	12,051 (0.6)
Smoking, 2022	1,808,420	369,775 (17.0)
Alcohol consumption, 2009	2,173,724	4,471 (0.2)
Alcohol consumption, 2022	1,763,079	415,116 (19.1)
Physical activity, 2009	2,177,754	441 (0.0)
Physical activity, 2022	2,177,754	441 (0.0)
Age at menarche	1,666,234	511,961 (23.5)
Age at menopause	1,312,015	866,180 (39.8)
Hormone replacement therapy	1,431,441	746,754 (34.3)
Parity	1,669,884	508,311 (23.3)
Breastfeeding history	1,626,496	551,699 (25.3)
Oral contraceptive use	1,670,474	507,721 (23.3)
Hypertension	2,177,754	441 (0.0)
Diabetes mellitus	2,177,754	441 (0.0)
Osteoporosis	2,177,754	441 (0.0)
Major depressive disorder	2,177,754	441 (0.0)
Sleep disturbance	2,177,754	441 (0.0)
Hearing loss	2,177,754	441 (0.0)

Note. BMI, body mass index. Missingness was assessed among 2,178,195 women remaining after eligibility and disease-related exclusions and removal of duplicate records, before listwise deletion for the complete-case analysis. The number of available observations and the number and percentage of missing observations are presented for each variable. Percentages may appear as 0.0% because of rounding.

Supplementary Table S3. Sensitivity analysis of factors associated with a broader composite cognitive and neurological outcome across five imputed datasets.

Factors	Category	OR across imputations	95% CI across imputations	p value
**Sociodemographic factors**				
Age	Per 1-year increase	1.10	1.099–1.110	<.001
	No change	Reference		
Change in economic status	Decreased	1.03	1.027–1.041	<.001
	Increased	1.00	0.991–1.005	<.001
	No change	Reference		
Change in employment status	Became unemployed	0.98	0.975–0.992	<.001
	Became employed	1.09	1.079–1.098	<.001
**Physiological factors**				
	No change	Reference		
Change in BMI category	Decreased	0.91	0.903–0.916	<.001
	Increased	1.06	1.049–1.063	<.001
	No change	Reference		
Change in hemoglobin level	<12 → ≥12 g/dL	1.00	0.992–1.009	<.001
	≥12 → <12 g/dL	0.88	0.876–0.890	<.001
	No change	Reference		
Change in total cholesterol level	Decreased	1.00	0.998–1.009	<.001
	Increased	0.98	0.974–0.990	<.001
**Lifestyle-related factors**				
Smoking	No change	Reference		
	Decreased	1.07	1.057–1.090	<.001
	No change	Reference		
Change in alcohol consumption	Decreased	1.01–1.02	0.997–1.027	.001–.024
	Increased	0.99	0.978–0.998	<.001–.005
	No change	Reference		
Change in physical activity	Adequate → insufficient	1.02	1.005–1.024	<.001
	Insufficient → adequate	1.07	1.062–1.075	<.001
**Reproductive factors**				
Age at menarche	Per 1-year increase	1.03	1.029–1.032	<.001
Age at menopause	Per 1-year increase	1.00	0.999–1.000	.060
Hormone replacement therapy	No	Reference		
	Yes	1.23–1.24	1.225–1.242	<.001
Parity	No	Reference		
	Yes	0.98–1.00	0.968–1.013	.26–.68
Breastfeeding history	No	Reference		
	Yes	1.06	1.049–1.071	<.001
Oral contraceptive use	No	Reference		
	Yes	1.06–1.07	1.050–1.067	<.001
**Comorbidity factors**				
Hypertension	No	Reference		
	Yes	1.02	1.015–1.026	<.001
Diabetes mellitus	No	Reference		
	Yes	1.17	1.162–1.175	<.001
Osteoporosis	No	Reference		
	Yes	1.56	1.555–1.572	<.001
Major depressive disorder	No	Reference		
	Yes	1.75	1.741–1.761	<.001

Factors	Category	OR across imputations	95% CI across imputations	p value
Sleep disturbance	No	Reference		
	Yes	1.55	1.540–1.557	<.001
Hearing loss	No	Reference		
	Yes	1.32	1.311–1.326	<.001

OR, odds ratio; CI, confidence interval; BMI, body mass index.

Note. As a sensitivity analysis, a broader composite cognitive and neurological outcome was defined to include MCI, dementia, Alzheimer's disease, cerebrovascular disease, Parkinson's disease, and other prespecified neurological disorders that were excluded from the primary analytic cohort. Five imputed datasets were analyzed separately using the same covariate set as in the primary imputation-specific analyses. Values are presented as the observed values or minimum-to-maximum ranges of ORs, 95% CIs, and *p* values across the five imputed datasets. Estimates were not pooled using Rubin's rules; therefore, these results should be interpreted descriptively rather than as pooled MI inference.
